# Discovery of a new mode of oviparous reproduction in sharks and its evolutionary implications

**DOI:** 10.1038/s41598-020-68923-1

**Published:** 2020-07-23

**Authors:** Kazuhiro Nakaya, William T. White, Hsuan-Ching Ho

**Affiliations:** 1 Hokkaido Univerisity, 3-1-1, Minato-cho, Hakodate, Hokkaido 041-8611 Japan; 2 CSIRO Australian National Fish Collection, National Research Collections Australia, GPO Box 1538, Hobart, Australia; 3 National Museum of Marine Biology and Aquarium, Pingtung, Taiwan; 4 Institute of Marine Biology, National Dong Hwa University, Pingtung, Taiwan

**Keywords:** Ecology, Evolution, Zoology

## Abstract

Two modes of oviparity are known in cartilaginous fishes, (1) single oviparity where one egg case is retained in an oviduct for a short period and then deposited, quickly followed by another egg case, and (2) multiple oviparity where multiple egg cases are retained in an oviduct for a substantial period and deposited later when the embryo has developed to a large size in each case. Sarawak swellshark *Cephaloscyllium sarawakensis* of the family Scyliorhinidae from the South China Sea performs a new mode of oviparity, which is named “sustained single oviparity”, characterized by a lengthy retention of a single egg case in an oviduct until the embryo attains a sizable length. The resulting fecundity of the Sarawak swellshark within a season is quite low, but this disadvantage is balanced by smaller body, larger neonates and quicker maturation. The Sarawak swellshark is further uniquely characterized by having glassy transparent egg cases, and this is correlated with a vivid polka-dot pattern of the embryos. Five modes of lecithotrophic (yolk-dependent) reproduction, i.e. short single oviparity, sustained single oviparity, multiple oviparity, yolk-sac viviparity of single pregnancy and yolk-sac viviparity of multiple pregnancy were discussed from an evolutionary point of view.

## Introduction

The reproductive strategies of the Chondrichthyes (cartilaginous fishes) are far more diverse than those of the other animal groups. Reproduction in chondrichthyan fishes is divided into two main modes, oviparity (egg laying) and viviparity (live bearing). Oviparity is restricted to the orders Carcharhiniformes (ground sharks), Heterodontiformes (bullhead sharks), Orectolobiformes (carpet sharks), Rajiformes (skates) and Chimaeriformes (chimaeras). Viviparity is prevailing in all chondrichthyan orders except in Heterodontiformes and Chimaeriformes whose members are all oviparous.

The morphology of members of the order Carcharhiniformes is less diverse compared to those of orders Lamniformes, Orectolobiformes and Squaliformes^1^, but this order is far more speciose, containing more species than in all the other eight shark orders combined. Moreover, the reproductive strategies of carcharhiniform sharks are more diverse than in any other chondrichthyan orders, having two modes of oviparity^1–7^ and three or four modes of viviparity^6,7^.

The catsharks (families Pentanchidae and Scyliorhinidae) are the largest group within the order Carcharhiniformes with about 150 species. Their reproduction is mostly oviparous, with several yolk-sac viviparous species. Among the oviparous catsharks, most of them perform single oviparity, in which one egg case is kept in each oviduct only for a short time and deposited soon after the egg case is completed. The other mode multiple oviparity is found in some catsharks, where several egg cases are retained in an oviduct for months prior to being deposited with a developing embryo in each egg case.

Recently, we found a new mode of oviparity in Sarawak swellshark *Cephaloscyllium sarawakensis* (family Scyliorhinidae, order Carcharhiniformes) from Taiwan that does not match the two currently known modes of oviparity, and it is herein termed “sustained single oviparity”. In addition, the egg cases of this species were found to be glassy transparent, which is quite unique among oviparous cartilaginous fishes. The main aims of present study are to describe the new mode of oviparity and transparent egg case, to propose a new set of technical terms for oviparity in the cartilaginous fishes, to discuss the importance of the new oviparity from the biological aspects of reproduction, and to refer to the evolution of lecithotrophic (yolk-dependent) reproduction in sharks.

## Results

### Egg cases and embryos

**Figure 1:**
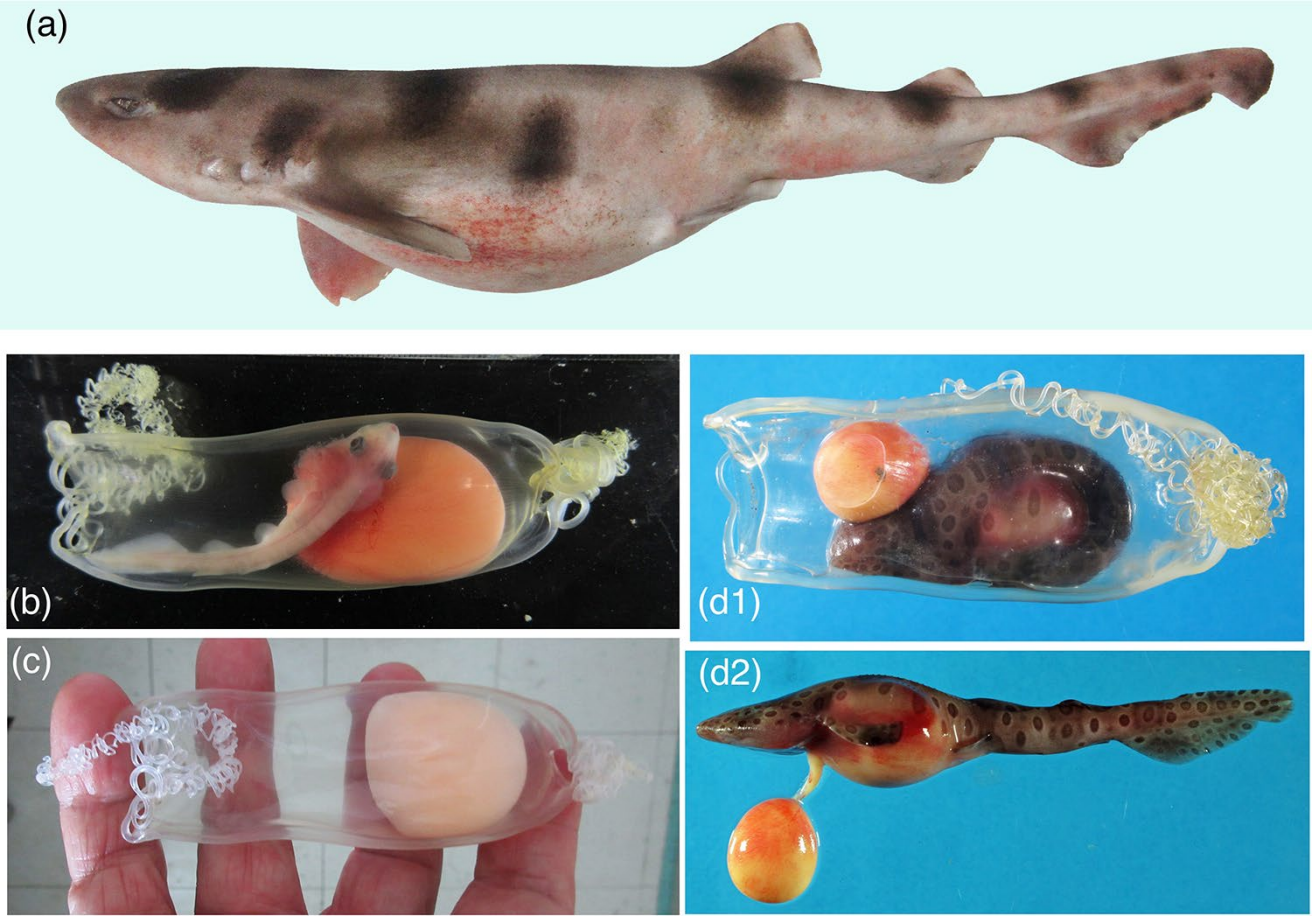
*Cephaloscyllium sarawakensis* and transparent egg cases. (**a**) 420 mm TL female with egg cases (NMMB-P 30853), (**b**) egg case and 64.8 mm TL embryo, from left oviduct, with external gills (NMMB-P30888, 81.6 mm ECL), (**c**) egg case from oviduct (NMMB-P30887, 79.9 mm ECL), showing transparency, (**d**) largest embryo 102 mm TL (NMMB-P 30991) in egg case (d1) and lateral view (**d2**).

All the pregnant specimens of Sarawak swellshark *Cephaloscyllium sarawakensis* examined (Fig. 1a) had one egg case in each oviduct, i.e. two egg cases in each individual. Egg cases examined were 75.0‒85.9 mm in length, excluding tendrils (ECL) (16.5‒20.1% TL) and 30.0‒39.5 mm in width (7.4‒8.7% TL) (Table 3), and occupied most of the available space of the oviduct. Egg cases had a smooth surface and with tough, thick shell, roughly quadrangular in shape and rather flattened, with a truncate anterior end and a rounded posterior end (Fig. 1b‒d). The egg case was completely transparent, and the yolk and embryo (when present) were clearly visible through the casing. A very long and strong tendril was present on the horn at four corners of the egg case. There were four openings, or corner slits, one at the base of each horn. Corner slits at anterior horns were linear and long, and those at posterior horns were curved. Corner slits were closed in the egg cases without developing embryo, and were open in the egg cases with 43 mm TL and larger embryos.

Eight egg cases from four females had only yolk, without developing embryos. The egg cases from nine females each contained a developing embryo. The largest embryo observed in the egg case (77.0 mm ECL) was 102 mm TL, with many polka-dot markings (Fig. 1d) and the smallest one was 43 mm TL without markings. External yolk was present on all these embryos.

**Figure 2:**
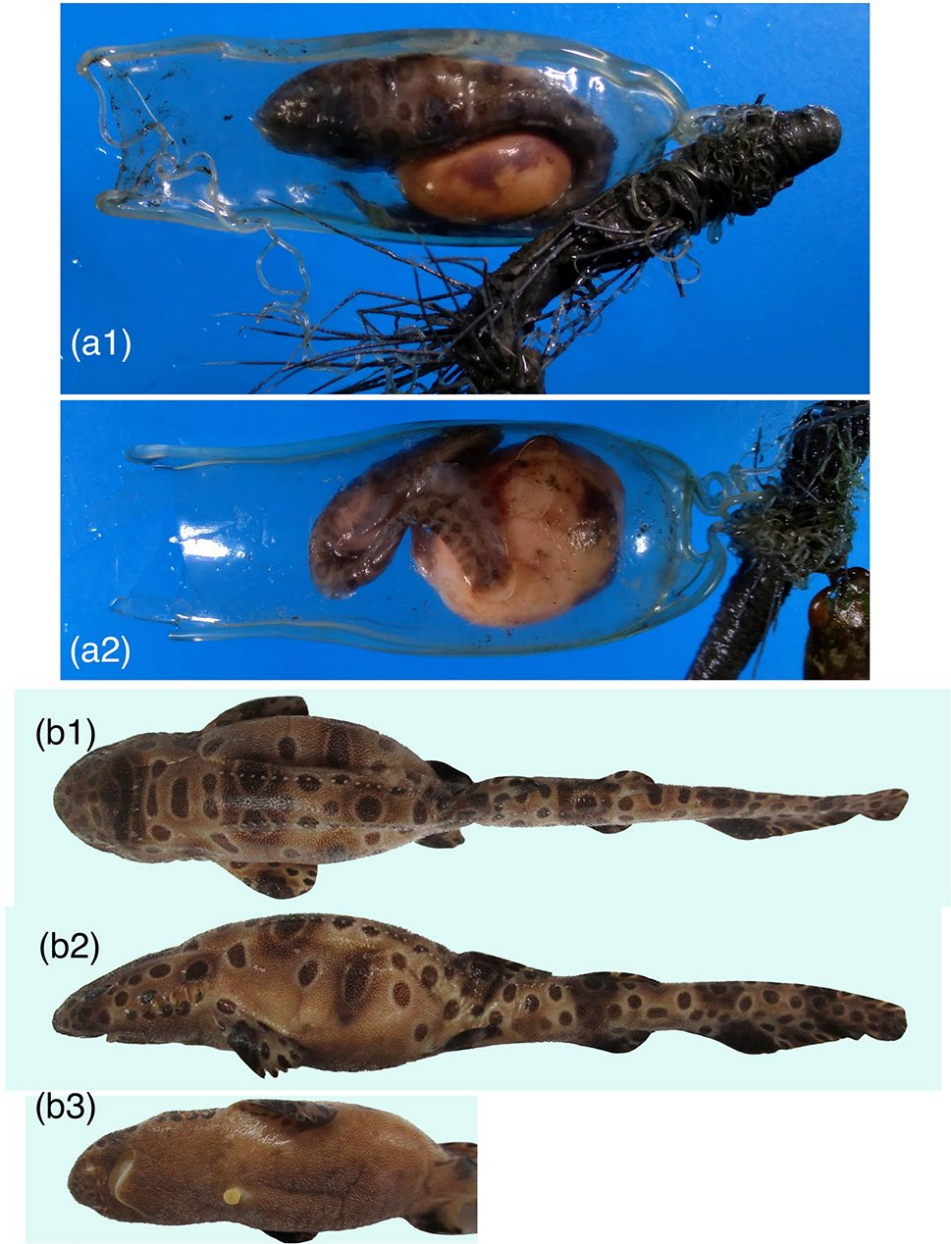
Egg case and embryo/juvenile of *Cephaloscyllium sarawakensis* collected by trawl net. (**a**) Egg cases laid on the tube of a tubeworm (a1 NMMB-P32797A, a2 NMMB-P32797B, both 80.0 mm ECL), (**b**) smallest juvenile (NMMB-P22719, 125 mm TL female) in dorsal (**b1**), lateral (**b2**) and latero-ventral view showing remnant of external yolk sac (**b3**).

Two already deposited egg cases were collected from trawl catch landings (Fig. 2a). One egg case (NMMB-P32979A, 80.0 mm ECL, Fig. 2a1) contained an embryo of 94.5 mm TL, and the other (NMMB-P32979B, 80.0 mm ECL, Fig. 2a2) had an embryo of 65.0 mm TL. Both embryos had dark polka-dot markings and external yolk that were clearly visible through the transparent casing. These egg cases were firmly tied to the tube of a tubeworm *Paradiopatra* sp. (family Onuphidae, order Eunicida) by posterior tendrils. The anterior tendrils of one egg case (NMMB-P32979A) were loosely entangled with the tube, and those of the other egg case (NMMB-P32979A) were broken and missing.

### Juveniles

The smallest, free-swimming specimen was a 125 mm TL female (NMMB-P22719) with a remnant of the external yolk sac (Fig. 2b). The body is light brownish with many dark polka-dot markings on lateral and dorsal surfaces of body. The second and third smallest juveniles were a 134 mm TL male (NMMB-P24872) and a 143 mm TL male (NMMB-P17143), both polka-dotted and with a reduced external yolk sac and a distinct scar.

## Discussion

**Figure 3:**
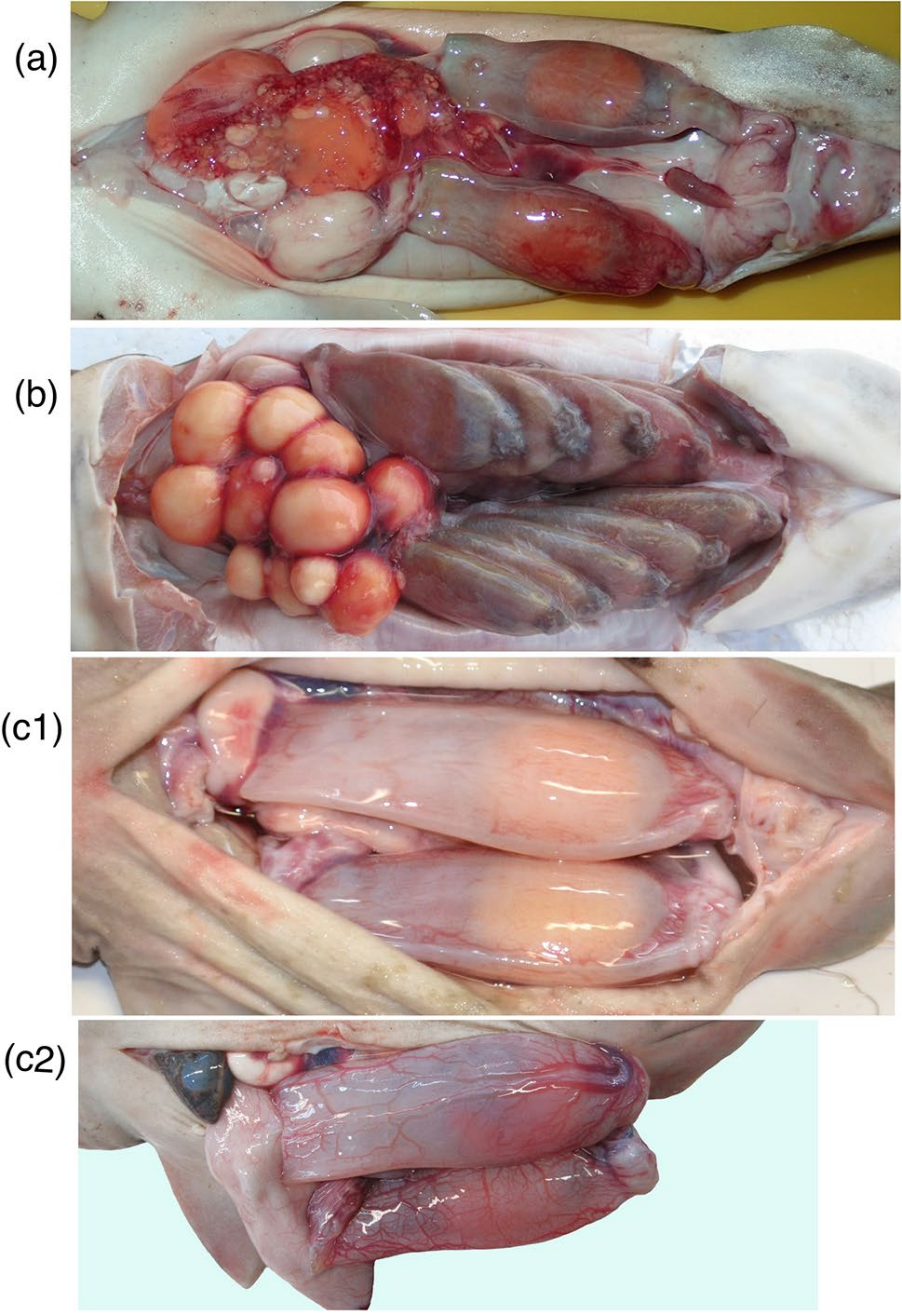
Three modes of oviparity in catsharks, showing egg cases in oviduct. (**a**) Short single oviparity (*Galeus sauteri* from Taiwan, uncatalogued), (**b**) multiple oviparity (*Halalelurus buergeri*, 410 mm TL from Kagoshima, Japan, uncatalogued), (**c**) Sustained single oviparity (*Cephaloscyllium sarawakensis*), (**c1**) egg cases without developing embryo (NMMB-P30890, 80.0 mm ECL), (**c2**) egg cases with a developing embryo in each (NMMB-P 30888, 81.6 mm, 83.0 mm ECL). Top three photographs (**a**,**b**,**c1**) cover whole abdominal cavities, showing difference of relative sizes of egg cases in three modes of oviparity. *Cephaloscyllium sarawakensis* (**c1**) has huge egg cases occupying most of the abdominal cavity.

**Figure 4:**
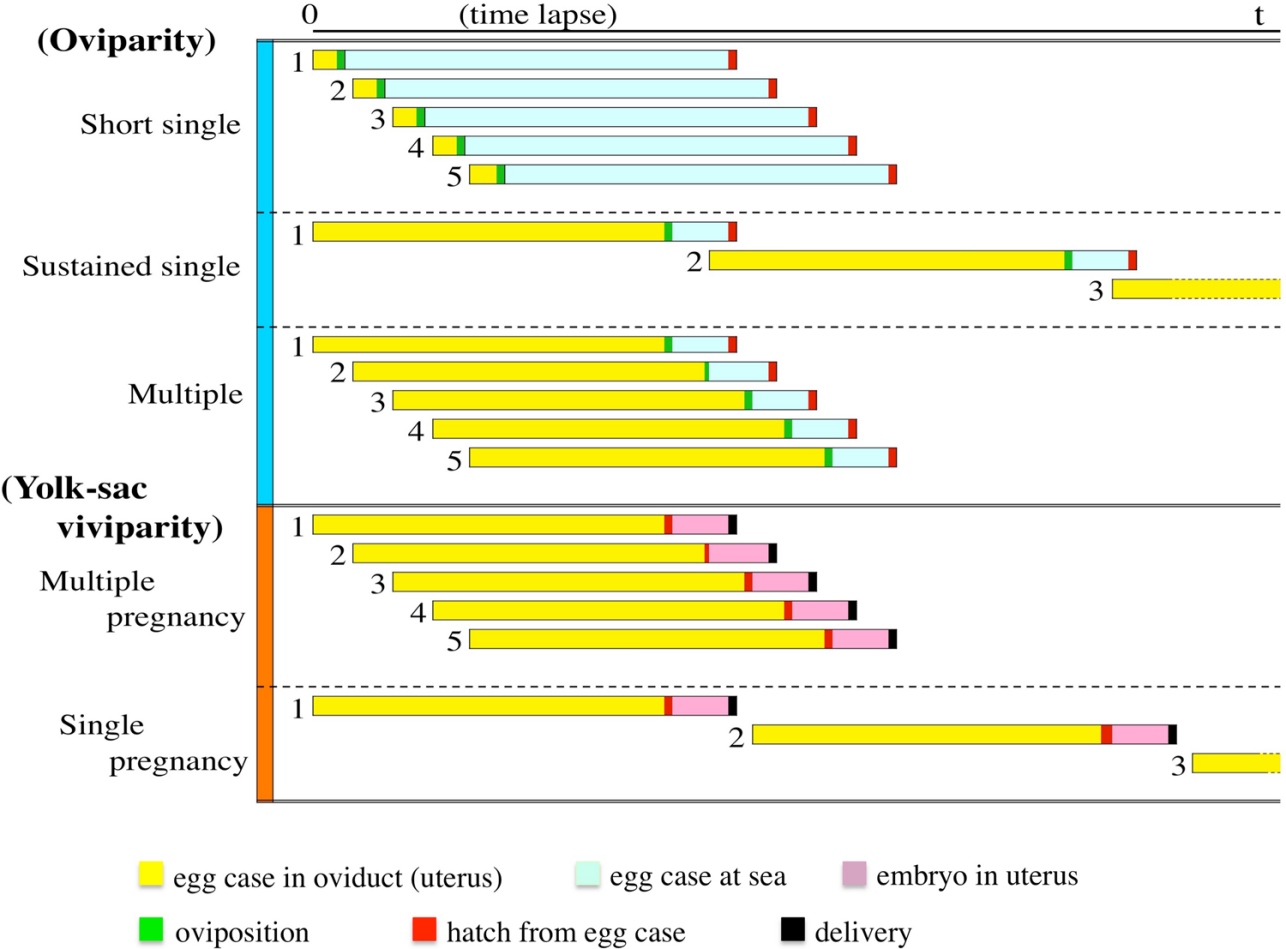
Schematic diagram of five modes of reproduction in catsharks, showing differences of succession, duration and condition of egg cases/ embryos per one oviduct/uterus. Numerals show order of egg case produced in the oviduct/uterus.

Two modes of oviparity, i.e. single and multiple oviparity, are currently recognized in chondrichthyans^1–3,5–12^. Single oviparity (Fig. 3a) is a mode where each oviduct in pregnant females contains one egg case, i.e. a pair of egg cases in a pregnant female. These egg cases are retained in the oviduct only for a short time and deposited immediately before the embryo has begun developing. The embryos are not recognizable at oviposition and become visible in a few weeks. Oviposition is repeatedly performed, and each mature female can deposit tens of egg cases over the course of a spawning season^6^ (“Short single” oviparity in Fig. 4). Multiple oviparity (Fig. 3b) is a mode where several egg cases accumulate in each oviduct and are retained for several months before oviposition, in which time embryos begin development in the oviduct and the egg cases are deposited later when the embryos grow large to a certain developmental stage (“Multiple” oviparity in Fig. 4).

*Cephaloscyllium sarawakensis* does not fit the classic single oviparity, nor multiple oviparity. Pregnant females of this species always have a single egg case, never two or more, in each oviduct (Fig. 3c), and keep it until embryo attains a certain developmental stage (“Sustained single” oviparity in Fig. 4). These facts indicate that reproduction of *C. sarawakensis* represents a new mode of oviparity, which is herein termed “sustained single oviparity”. A 450 mm TL female of *Cephaloscyllium silasi* from the Indian Ocean had one egg case with a well-developed embryo in each oviduct^13^. Although they reported only one female specimen, this species possibly also displays the sustained single oviparity.

Various technical terms have been used for oviparity. Single oviparity has at least three alternative names, “extended” oviparity^3,4,6,12,14^, “external” oviparity^5,15^, and “simple” oviparity^5^. Multiple oviparity has been also termed “retained” oviparity^3–6,12,14,16^. These terms are used for the same reproductive mode, and this could lead to confusion and misunderstanding about the oviparity. Therefore, we herein summarized the terms of oviparity and proposed a new set of technical terms as follows: (1) “short single oviparity” for the single oviparity previously known; (2) “sustained single oviparity” for the new type of oviparity reported in this study; and (3) “multiple oviparity” instead of the former “retained” oviparity. The new definition of oviparity in the cartilaginous fishes was summarized, with additions of two modes of yolk-sac viviparity in catsharks (Table 1).

**Table 1:** New definitions of oviparity and yolk-sac viviparity in lecithotrophic (yolk-dependent) cartilaginous fishes.

Parity	Mode proposed	Definition	Order	Family	Genus and/or species	Sources
OVIPARITY^a^	Short single oviparity (syn. short, extended, external and simple oviparity)	One egg case per oviduct is kept for a short time, and is laid immediately before embryo develops	Carcharhiniformes	Pentanchidae	*Apristurus*, *Asymbolus*, *Figaro*, *Haploblepharus*, *Holohalaelurus*, *Parmaturus*	^1,2,37,39,56^
					*Bythaelurus bachi*, *B. canescens*, *B. dawsoni*, *B. naylori*, *B. vivaldi*	^28,38,53^
					*Galeus antilensis*, *G. arae*, *G. cadenati*, *G. eastmani*, *G. mincaronei*, *G. murinus*, *G. nipponensis*, *G.sauteri*	^1,2,8,25,35,57^
				Scyliorhinidae	*Atelomycterus*, *Cephaloscyllium* (excluding *C. sarawakensis* and *C. silasi*), *Poroderma*, *Schroederichthys*, *Scyliorhinus*	^1,2,39^
				Proscylliidae	*Proscyllium*	^1,39,present paper^
			Orectolobiformes	Parascylliidae	*Cirrhoscyllium*, *Parascyllium*	^4^
				Hemiscylliidae	*Chiloscyllium*, *Hemiscyllium*	^4^
			Heterodontiformes	Heterodontidae	*Heterodontus*	^1,39,present paper^
			Rajiformes		All genera in Arhynchobatidae, Anacanthobatidae, Rajidae	^58^
			Chimaeriformes		all genera	
	Sustained single oviparity	One egg case per oviduct is retained for a long time, and is laid after embryo grows large	Carcharhiniformes	Scyliorhinidae	*Cephaloscyllium sarawakensis*, *C. silasi*	^13,present paper^
	Multiple oviparity (syn. retained oviparity)	Plural egg cases per oviduct are retained for a long time, and are laid after embryos grow large	Carcharhiniformes	Pentanchidae	*Halaelurus boesemani*, *H. buergeri*, *H. lineatus*, *H. maculosus*, *H. natalensis*, *H. quagga*, *H. sellus*	^1,25,33–35,59,60^
					*Galeus atlanticus*, *G. melastomus*, *G. piperatus*	^30,39,61^
			Orectolobiformes	Stegostomatidae	*Stegostoma fasciatum*	^4^
VIVIPARITY^b^	yolk-sac viviparity (single pregnancy)	One embryo per uterus is retained until delivery	Carcharhiniformes	Pentanchidae	*Bythaelurus clevai*, *B. hispidus*, *B. lutarius*, *B. stewarti*	^37,38,53^ ^1^
					*Cephalurus cephalus*	
	yolk-sac viviparity (multiple pregnancy)	Plural embryos per uterus are retained until delivery	Carcharhiniformes	Pentanchidae	*Galeus polli*	^1^

^a^Oviparous genus *Aulohalaelurus* (Scyliorhinidae, Carcharhiniformes) is excluded, because the mode is not determined yet.

^b^Only catsharks are given.

**Figure 5:**
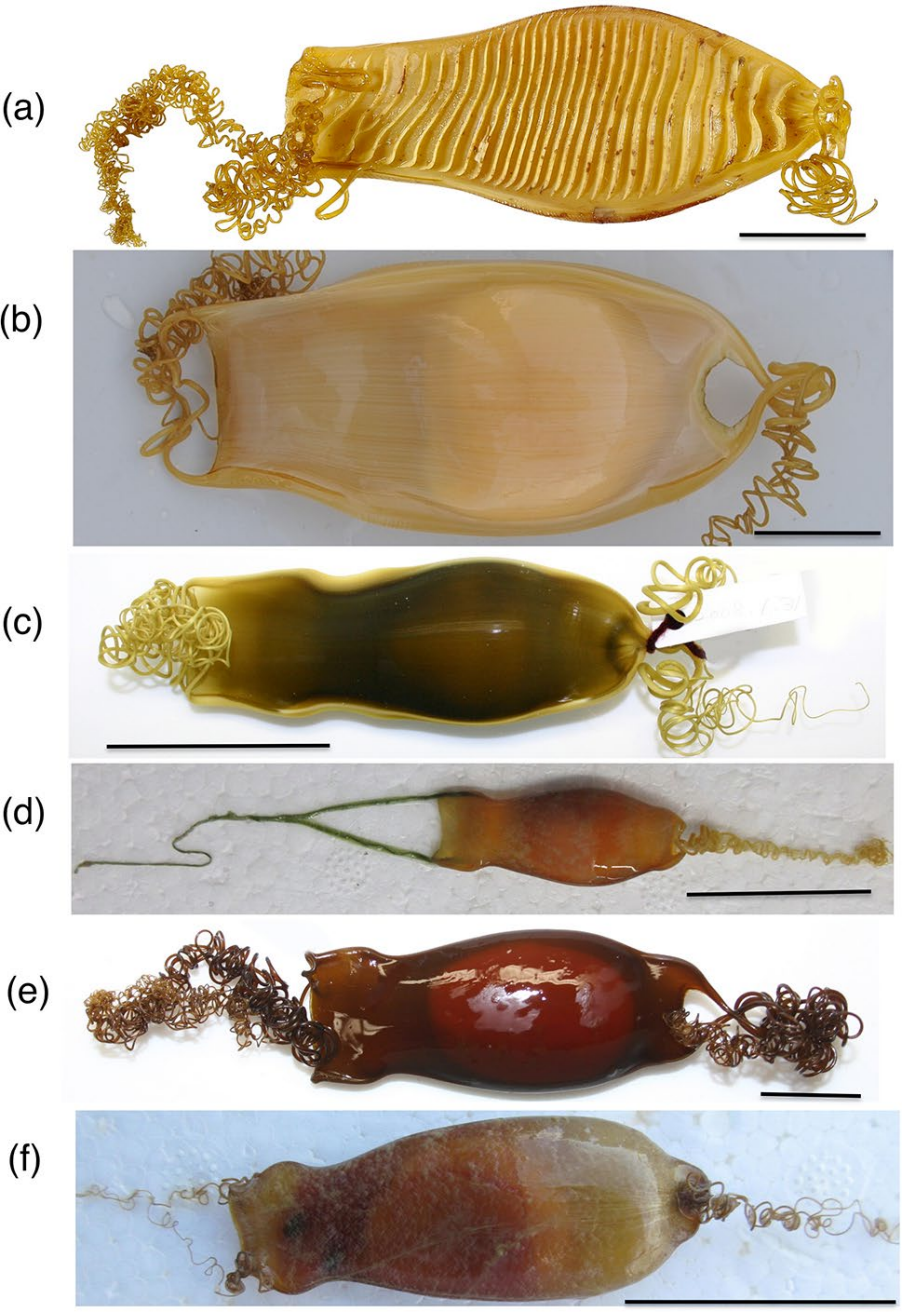
Egg cases of catsharks. (**a**) *Cephaloscyllium laticeps* from Australia, (**b**) *Cephaloscyllium umbratile* from Japan, (**c**) *Poroderma africanum* from Ibaraki Prefectural Oarai Aquarium, Japan, (**d**) *Galeus sauteri* from Taiwan, (**e**) *Haploblepharus fuscus* from Ibaraki Prefectural Oarai Aquarium, Japan, (**f**) *Halaelurus buergeri* from Japan. (**a**‒**e**) short single oviparous species, (**f**) multiple oviparous species. Scales 30 mm.

Typically in the catsharks displaying short single oviparity, the shell of egg case is tough and thick, with two pairs of long strong tendrils on its anterior and posterior ends (Fig. 5a‒c,e,f), although tendrils are very short or replaced by fine silky materials (Fig. 5d) or absent in some species. The posterior pair of tendrils is used to attach the egg case to the substrate on the sea bottom, to pull it out from the oviduct, and coil it around the substrate, together with anterior pair of tendrils. The egg case is firmly secured on the substrate until juvenile hatches. The tendrils in the multiple oviparous species (Fig. 5f) tend to be thinner and shorter than those of the short single oviparous species. *Cephaloscyllium sarawakensis* of the sustained single oviparity has a thick shell and long tendrils (Fig. 1b,c,d1). The two egg cases (Fig. 2a) collected from fishery landings can be inferred to have been deposited intentionally on the tube of a tubeworm *Paradiopatra* sp. based on the fact that the posterior tendrils are firmly twined around it.

The egg cases in the species of sustained single oviparity are very large, with its length (ECL) ranging 16.5‒20.1% TL in *Cephaloscyllium sarawakensis* and 18.9‒19.2% TL in *C. silasi*^13^, while the egg cases of short single oviparous *C. umbratile* are 10.6‒15.1% TL^2,17,18^. The egg cases of other short single oviparous species are also small, with lengths 8.6‒9.0% TL in *Holohalaelurus regani*^19^, 9.2% TL in *Galeus sauteri*^present study^, 9.2‒14.8% TL in four species of *Apristurus*^20–23^, 10.6‒14.9% TL in *Atelomycterus marmoratus*^24^, 11.4% TL in *Schroederichthys maculatus*^25^, 11.5‒11.9% TL in *Scyliorhinus torazame*^26,presentstudy^, 11.8% TL in *S. capensis*^27^, 12.0% TL in *Bythaelurus dawsoni*^28^ and 13.5‒18.9% TL from Fig. 10d^29^ of *Parmaturus xaniurus*. The egg case lengths of multiple oviparous species are about 11% TL in *Halaelurus buergeri*^present study^ and 10.4‒10.9% TL in *H. quagga*^30^. As seen in Fig. 3, the egg cases of *C. sarawakensis* (Fig. 3c1) are far larger than those of *G. sauteri* (Fig. 3a) and *H. buergeri* (Fig. 3b), occupying most of the available space of oviduct and abdominal cavity. Thus, the two species of sustained single oviparity have much larger egg cases than short single oviparous and multiple oviparous species, suggesting larger neonates at hatching in *C. sarawakensis* and *C. silasi*.

The embryos in the egg cases recorded from the oviduct indicate that *Cephaloscyllium sarawakensis* retains the egg case until the embryo attains about 102 mm TL (Fig. 1d; largest embryo in the egg inside the oviduct), and then oviposition occurs later. However, the two egg cases (both 80.0 mm ECL) from the seabed contained a 65 mm TL and a 94.5 mm TL embryo each (Fig. 2a). These facts suggest the timing of oviposition is rather wide in this species, and the egg cases are laid when the embryo grows roughly 6‒10 + cm TL, stimulated by some internal or external factors. The smallest free-swimming juvenile collected was 125 mm TL with a remnant of external yolk-sac (Fig. 2b), suggesting the hatching size from egg case being around 120 mm TL in this species. These evidences indicate *C. sarawakensis* keeps the egg case in the oviduct until embryo has developed to 50 ~ 80+ % of its hatching size. Therefore, the retention of egg case in the oviduct continues for an extended period, perhaps several months or more, in *C. sarawakensis* and probably also in *C. silasi*.

The other remarkable characteristic of *Cephaloscyllium sarawakensis* is the glassy transparent egg cases (Fig. 1b‒d), and the transparency is completely maintained even after oviposition (Fig. 2a). The egg cases of oviparous cartilaginous fishes are opaque, usually yellowish to dark brownish (Fig. 5), and sometimes with longitudinal or transverse ridges (Fig. 5a). The functional role of egg case is to protect the embryo from physical, physiological and biological hazards from the environment. The colored egg cases can be also effective to conceal the embryo in it. However, the egg case of *C. sarawakensis* is transparent and never cryptic that the orange yellow yolk would clearly be recognizable through transparent egg case, if the egg case is deposited immediately after egg case being formed.

As shown in this study, *C. sarawakensis* retains an egg case in each oviduct until the embryo is developed with a distinct dark polka-dot color pattern on light brownish body (Figs. 1d, 2a), typically seen in the juveniles (Fig. 2b). One of the reasons for transparent egg case may be related to their vivid body color patterns. Benthic and reef-dwelling sharks have complicated color patterns, which are effective to blend the body into their background or for camouflage^31^. The present egg cases of *C. sarawakensis* (Fig. 2a) were deposited around the tube of a tubeworm sticking out from the seabed. Their vivid polka-dots and light brownish body coloration could function as more effective camouflage against the complex and dark background through the transparent egg case. Thus, the long retention of transparent egg cases and vivid embryonic coloration could suggest a new method of reproductive tactics in cartilaginous fishes.

The oviparity is advantageous as a method to increase the fecundity in small elasmobranchs that have limited space in body cavity for care and storage of the embryos^6^. The species of short single oviparity (Fig. 3a) repeatedly deposits two egg cases immediately after the cases are completed and laid, resulting in 20‒100 eggs per season^6^. The captive *Cephaloscyllium umbratile* was recorded as depositing two egg cases at intervals of 11‒38 days (20 days in average) for whole year^32^, which means a single female deposited about 36 egg cases a year. Similarly, the captive *C. laticeps* laid two egg cases at intervals of up to 28 days throughout whole year^11^, equating more than 26 egg cases being deposited annually.

The multiple oviparous species retains a number of egg cases in each oviduct (Fig. 3b) for several months until the embryos have developed to a certain stage. All the species of *Halaelurus* are multiple oviparous, and *H. buergeri* has been recorded to deposit 8 egg cases one by one at a stage when the embryos inside have attained 70 mm TL^33^, or 10 egg cases at one time^34^. One specimen of *H. buergeri* we (KN) collected (Fig. 3b) had 10 egg cases in the oviducts with a developing embryo in each. A captive *H. maculosus* deposited 11 egg cases containing 50‒70 mm TL embryos in five days (personal communication with Mr. K. Tokunaga of Ibaraki Prefectural Oarai Aquarium). The fecundity of *H. lineatus* is up to 16 eggs at a time^35^.

The maternal environment offers the best protection to the developing embryos and can shorten the exposed period of time on the substrate. Therefore, the survival rate could be expected to be much higher in the sustained single and multiple oviparous species than the short single oviparous species. The species of the sustained single oviparity (Fig. 3c) and those of the multiple oviparity (Fig. 3b) are the same in that the egg cases have long maternal protection, but the number of the egg cases deposited at a time is considerably less in the sustained single oviparous species, i.e. 2 eggs vs. 4‒16 eggs per mother, respectively. Hence, the fecundity of *Cephaloscyllium sarawakensis* could be very low, 1/8–1/2 of the multiple oviparous species (see “Oviparity” in Fig. 4).

It is crucial to produce a certain number of offspring to maintain a sustainable population, and the very low fecundity in *C. sarawakensis* could be decisively disadvantageous for the species. Similar issues exist in the yolk-sac viviparous species. The yolk-sac viviparous catsharks, such as *Bythaelurus clevai*, *B. hispidus, B. lutarius*, *B. stewarti* and *Cephalurus cephalus* retain only one embryo per uterus or per mother^1,28,36–38,^^KN pers.obs^, which is hence termed here “single pregnancy”. These species of single pregnancy would have also lower fecundity than the species of “multiple pregnancy” seen in *Galeus polli* (see “Yolk-sac viviparity” in Fig. 4).

Species of the genus *Cephaloscyllium* are generally large in body sizes, mostly growing to more than 70 cm TL and some species (*C. isabellum, C. laticeps, and C. umbratile*) attain more than 100 cm TL^39^, whereas *C. sarawakensis* and *C. silasi* are dwarf species within the genus. *Cephaloscyllium sarawakensis* attains a maximum of only 39.7 cm TL in males and 49.5 cm TL in females^40,present study^, and matures at the sizes less than 32.5 cm TL and 35.4 cm TL in males and females, respectively^41^. Similarly, *Cephaloscyllium silasi* attains only 50 cm TL^13^ and reaches its maturity at less than 36.8 cm TL in males^1^ and less than 45 cm TL in females^13^. *Bythaelurus clevai, B. hispidus* and *B. lutarius* attain 42 cm TL, 36 cm TL and 39 cm TL, respectively^37,39^ and these species are also the smallest species for the genus. *Cephalurus cephalus* reaches 30 cm TL^36,39^, and this is known as one of the smallest sharks^1^.

The ratio of length at maturity to the largest total length was reported at around 0.73 for elasmobranchs^42^, and this indicates the smaller species could reach their maturity at smaller sizes than the larger species. Actually, the captive *Cephaloscyllium umbratile* which grows up to 118 cm TL hatches at lengths of 16–22 cm TL^32^ and attains its full maturity at 96‒104 cm TL^17^. In contrast, *C. sarawakensis* which produces very large egg cases relative to the mother size is expected to hatch at about 12 cm TL and mature at less than 35 cm TL. Therefore, *C. sarawakensis* grows only about 23 cm in length until maturity is attained, whereas *C. umbratile* needs to grow 75–90 cm to its maturity. *Cephalurus cephalus* produces 7–9 cm TL neonates and attains maturity at 18–22 cm TL^43^, thus 9–15 cm in length to grow to attain maturity. *Eridacnis radcliffei* gives birth 10.5‒12.8 cm TL neonates^36^, and attains maturity at 18.3 cm TL, only 5.5‒7.8 cm in length to the maturity.

Hence, these dwarf sharks of the sustained single oviparity and the yolk-sac viviparity of single pregnancy likely attain their maturity in a shorter time frame than the larger species do, enabling them to reproduce at an earlier age and keep their life-time fecundity high. Other factors to increase their lifetime fecundity include quick repetition of reproduction, longer lifetime reproduction and higher proportion of females, but these will not be referred to here.

**Figure 6:**
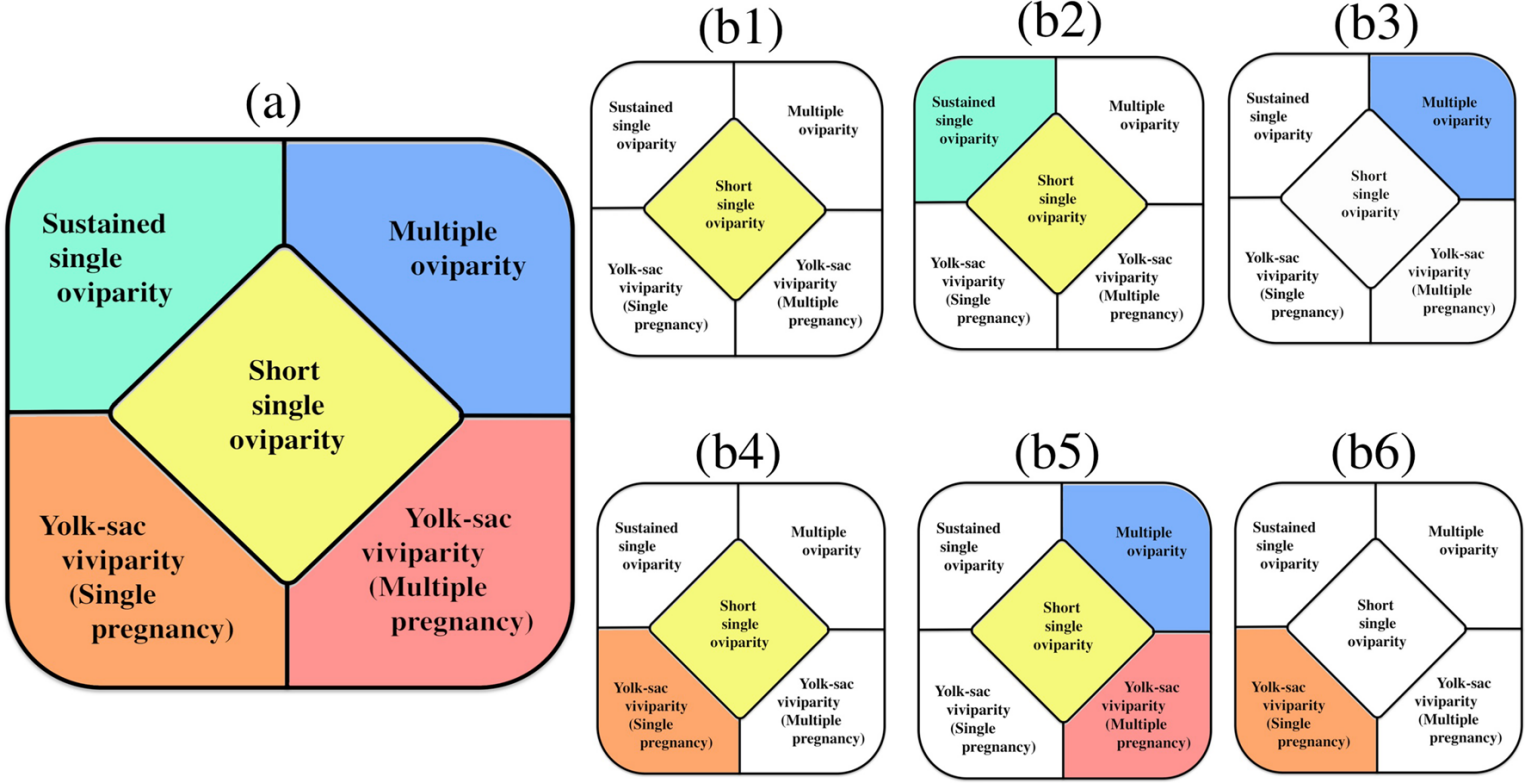
Modes of reproduction (**a**) and combination of the modes at generic level in catsharks (**b**). (**a**) Five modes of lecithotrophy (yolk-dependent reproduction) in cartilaginous fishes, (**b1**) *Apristurus*, *Asymbolus*, *Atelomycterus*, *Figaro*, *Haploblepharus*, *Holohalaelurus*, *Parmaturus, Poroderma, Scyliorhinus* and *Schroederichthys*, (**b2**) *Cephaloscyllium*, (**b3**) *Halaelurus*, (**b4**) *Bythaelurus*, (**b5**) *Galeus*, and (**b6**) *Cephalurus*.

Figure 6a shows five modes of lecithotrophic (yolk-dependent) reproduction in the cartilaginous fishes, and Fig. 6b1–6 denote six combinations of these reproductive modes in the catsharks. Figure 6b1 (short single oviparity) represents ten genera such as *Apristurus*, *Asymbolus*, *Atelomycterus*, *Figaro*, *Haploblepharus*, *Holohalaelurus*, *Parmaturus, Poroderma, Scyliorhinus* and *Schroederichthys*. Figure 6b3 (multiple oviparity) and Fig. 6b6 (yolk-sac viviparity of single pregnancy) are *Halaelurus* and *Cephalurus*, respectively. However, Fig. 6b2,b4,b5 include two or three modes of reproduction. *Cephaloscyllium* (Fig. 6b2) performs short single oviparity + sustained single oviparity, *Bythaelurus* (Fig. 6b4) involves short single oviparity + yolk-sac viviparity of single pregnancy, and *Galeus* (Fig. 6b5) performs short single oviparity + multiple oviparity + yolk-sac viviparity of multiple pregnancy. These facts could suggest rather facile diversification of reproductive mode in closely related species, or may suggest necessity of some taxonomic reconsideration of them, as indicated for *Bythaelurus*^38^.

Oviparity has been suggested to be the ancestral mode of reproduction for vertebrates^7,44^, and it has also traditionally been believed as the ancestral mode for chondrichthyan fishes^3,14,45^. However, recent studies^5,6,12,46,47^ suggest that viviparity is ancestral for all chondrichthyans, with many reversions to oviparity and secondary reversions to viviparity. Phylogenetic interrelationships for the Galeomorphi (orders Heterodontiformes, Orectolobiformes, Lamniformes and Carcharhiniformes)^4,45,48^^‒^^50^ show that short single oviparity is the ancestral mode for the Galeomorphi, and also for the orders Heterodontiformes, Orectolobiformes and Carcharhiniformes. Multiple oviparity is generally considered to have evolved from short single oviparity^5^, or evolved intermediately between the short single oviparity and the yolk-sac viviparity^1,7,14,51^.

**Figure 7:**
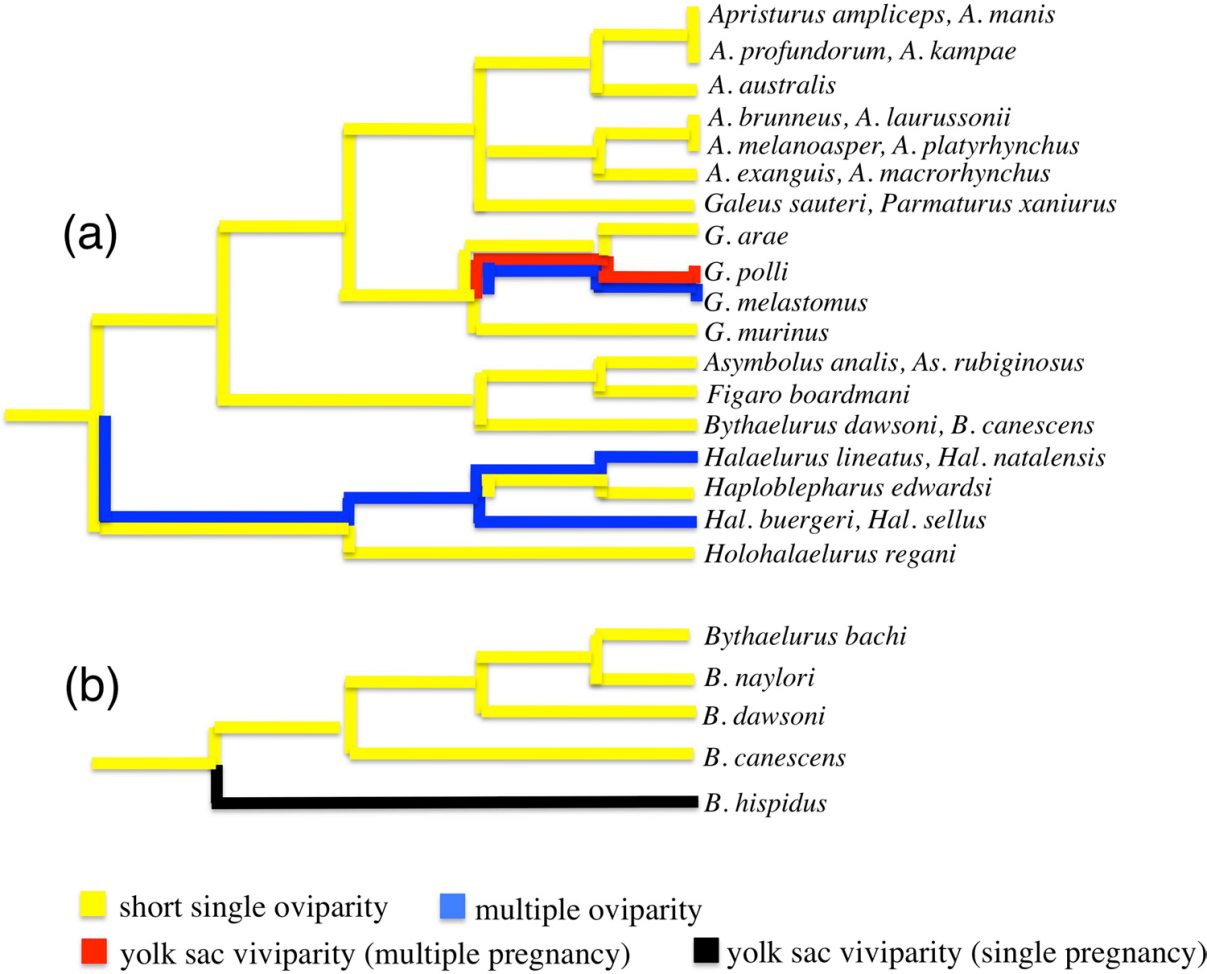
Reproductive modes mapped on simplified relationship of (**a**) Scyliorhinidae I^50^ and (**b**) *Bythaelurus*^53^, and suggested evolution.

The catsharks (now Pentanchidae and Scyliorhinidae) in the Carcharhiniformes are separated into a few isolated groups, based on genetic works^49,50,52^. Mapping of the reproductive modes on their phylogenetic relationships suggests that the short single oviparity is ancestral for each group. According to the phylogenetic result^50^ which covers more catshark taxa than the other works, their Scyliorhinidae I^50^ (Fig. 7a) includes nine genera of the family Pentanchidae. Six genera of these, *i.e. Apristurus*, *Asymbolus*, *Figaro*, *Haploblepharus*, *Holohalaelurus* and *Parmaturus*, display short single oviparity. The multiple oviparous genus *Halaelurus* is sister to the groups of short single oviparous catsharks, and the relationships suggest the multiple oviparity has derived from the short single oviparity.

The genus *Bythaelurus*, which is also deeply merged in groups of short single oviparous species in the Scyliorhinidae I^50^ (Fig. 7a), is currently comprised of 14 species^38,53,54^, with five short single oviparous species (*B. bachi, B. canescens, B. dawsoni, B. naylori* and *B. vivaldi*) and four yolk-sac viviparous species of single pregnancy (*B. clevai*, *B. hispidus, B. lutarius* and *B. stewarti*) (Fig. 6b4). Interrelationships of five *Bythaelurus* species^53^ (Fig. 7b) show they are clearly separable in two groups, i.e. short single oviparous species (*B. bachi, B. naylori, B. dawsoni* and *B. canescens*) and yolk-sac viviparous species of single pregnancy (*B. hispidus*). The short single oviparous *B. dawsoni* and *B. canescens* have a sister relation with short single oviparous genera *Asymbolus* + *Figaro* in the Scyliorhinidae I^50^ (Fig. 7a). These facts suggest that the short single oviparity is ancestral for *Bythaelurus* and the yolk-sac viviparity of single pregnancy could have derived from the short single oviparity^1^, maybe via sustained single oviparity.

The genus *Galeus* contains 18 species with three reproductive modes (Fig. 6b5), i.e. short single oviparity (*G. antillensis* and seven other species), multiple oviparity (*G. atlanticus*, *G. melastomus and G. piperatus*) and yolk-sac viviparity of multiple pregnancy (*G. polli*). The genus *Galeus* is deeply embedded within groups of short single oviparous species in the Scyliorhinidae I^50^ (Fig. 7a), and the short single oviparity is considered to be ancestral for the genus *Galeus*, and the yolk-sac viviparity of multiple pregnancy in *G. polli* could have derived from short single oviparity via multiple oviparity.

Their Scyliorhinidae II^50^ includes three genera *Atelomycterus*, *Schroederichthys* of short single oviparity and oviparous *Aulohalaelurus*^55^. Scyliorhinidae III^50^ includes three genera *Cephaloscyllium*, *Scyliorhinus* and *Poroderma*, and they all display short single oviparity. *Cephaloscyllium sarawakensis* and *C. silasi* of sustained single oviparity were not treated in their analysis^50^, but the relationships of *Cephaloscyllium* in the Scyliorhinidae III^50^ that is composed of short single oviparous species could suggest derivation of the sustained single oviparity directly from short single oviparity by longer retention of one egg case in an oviduct.

The modes of reproduction in the catsharks were summarized in Table 2, and the phylogenetic evidences mentioned above suggest: (1) short single oviparity is ancestral for the catsharks; (2) more diverse modes of reproduction evolved in the family Pentanchidae than family Scyliorhinidae; (3) sustained single oviparity in *Cephaloscyllium* was derived directly from short single oviparity; (4) multiple oviparity in *Halaelurus* was derived from short single oviparity; (5) multiple oviparity in *Galeus* was derived from short single oviparity, and originated yolk-sac viviparity of multiple pregnancy; (6) yolk-sac viviparity of single pregnancy in *Bythaelurus* was derived from short single oviparity, possibly via sustained single oviparity; and (7) yolk-sac viviparity of single pregnancy in *Cephalurus* was derived possibly from short single oviparity via sustained single oviparity.

**Table 2:** Modes of reproduction and evolution in catsharks.

Parity	Mode	Pentanchidae					Scyliorhinidae	
		*Apristurus*, *Asymbolus*, *Figaro*, *Haploblepharus*, *Holohalaelurus*, *Parmaturus*	*Bythaelurus*	*Galeus*	*Halaelurus*	*Cephalurus*	*Atelomycterus*, *Poroderma*, *Scyliorhinus*, *Schroederichthys*	*Cephaloscyllium*
Oviparity^a^	Short single	⦿	⦿	⦿	○	○	⦿	⦿
	Sustained single		○			○		⦿
	Multiple			⦿	⦿			
Yolk sac viviparity	Single pregnancy		⦿			⦿		
	Multiple pregnancy			⦿				

⦿Actual mode, ○ possible ancestral or transitional mode.

^a^Oviparous *Aulohalaelurus* in the Scyliorhinidae is not included, because the mode is unknown.

### Materials and methods

All specimens of *Cephaloscyllium sarawakensis* examined were bycatch from commercial bottom trawlers operating in the South China Sea off southwest Taiwan, and were collected at Hsin-da port (HD) and Ke-tzu-liao (KTL) in Kaohsiung. Specimens were fixed in 4% formalin and then transferred to 70% Ethanol or 50% Isopropanol ethanol. All specimens were deposited at Pisces collection of the National Museum of Marine Biology & Aquarium, Pingtung, Taiwan (NMMB-P). Total length (TL) was measured using a ruler or digital caliper, to nearest 1 or 0.1 mm, respectively.

Egg cases (Table 3): a total of 8 egg cases without visible embryo on the yolk, and 15 egg cases with a developed embryo each were collected from the oviduct of thirteen specimens of *C. sarawakensis*. Two egg cases with a developed embryo tied on the tube of a tubeworm *Paradiopatra* sp. (family Onuphidae, order Eunicida) were collected from the fishery landings by fishers, and were kept frozen. Egg cases were deposited and catalogued in NMMB-P collection. Length (excluding tendrils, ECL) and width (ECW) of the egg case were measured by a ruler or digital caliper.

**Table 3:** Measurements of egg cases and embryos, and sampling data of *Cephaloscyllium sarawakensis*.

NMMB-P	Mother (mm TL)	Egg case from	Embryo	Egg case length		Egg case width		Locality	Date
			mm TL	mm ECL	%TL	mm ECW	%TL		
30890	405	Right oviduct	0	80.0	19.8	30.0	7.4	KTL	2015/6/11
		Left oviduct	0	80.0	19.8	31.0	7.7		
30883	440	Right oviduct	0	75.0	17.0	35.0	8.0	HD	2019/3/19
		Left oviduct	0	75.0	17.0	34.0	7.7		
30887	443	Right oviduct	0	79.9	18.0	34.5	7.8	HD	2019/3/19
		Left oviduct	0	80.4	18.1	35.7	8.1		
30965	455	Right oviduct	0	79.6	17.5	35.5	7.8	HD	N/A
		Left oviduct	0	75.0	16.5	35.3	7.8		
30888	420	Right oviduct	61.7	83.0	19.8	33.0	7.9	HD	2019/3/19
		Left oviduct	64.8	81.6	19.4	33.9	8.1		
30968	420	Right oviduct	61.5	82.1	19.5	36.7	8.7	HD	N/A
		Left oviduct	68.4	84.4	20.1	36.4	8.7		
30884	435	Right oviduct	46.0	75.0	17.2	33.0	7.6	HD	2019/3/19
		Left oviduct	43.0	76.0	17.5	36.0	8.3		
30970	439	Right oviduct	66.6	85.1	19.4	35.7	8.1	HD	N/A
		Left oviduct	65.4	84.1	19.4	34.8	7.9		
30969	452	Right oviduct	63..3	80.0	17.7	33.9	7.5	HD	N/A
		Left oviduct	64.2	80.2	17.7	35.3	7.8		
30967	455	Right oviduct	57.6	82.4	18.1	36.2	8.0	HD	N/A
		Left oviduct	65.6	83.0	18.2	37.4	8.2		
30851	495	Right oviduct	not collected	N/A	N/A	N/A	N/A	HD	2019/3/19
		Left oviduct	70.6	85.9	17.4	39.5	7.9		
30819	Not collected	Right oviduct	68.0	82.0	N/A	34.0	N/A	KTL	2019/1/11
		Left oviduct	not collected	N/A	N/A	N/A	N/A		
30991	Not collected	Right oviduct	102	77.0	N/A	33.0	N/A	KTL	N/A
		Left oviduct	not collected	N/A	N/A	N/A	N/A		
32979A	Unknown	Seabed	94.5	80.0	N/A	30.0	N/A	KTL	2020/1
32979B	Unknown	Seabed	65.0	80.0	N/A	30.0	N/A	KTL	2020/1

*KTL* Ketzuliao, Kaohsiung, south western Taiwan, *SD* Hsinda Port, Kaohsiung, south western Taiwan.

Juveniles: NMMB-P22719, 125 mm TL female, KTL, 2 Apr. 2015; NMMB-P17143, 143 mm TL male; NMMB-P24872 (1 of 6 specimens), 134 mm TL male, KTL, 18 Mar. 2016.
